# Auto-Intoxication

**Published:** 1903-02-07

**Authors:** 


### AUTO-INTOXICATION.

Recent research has gone far to emphasise the importance of the rôle of toxic agents in the production of disease. Many of these are introduced into the system from without, and concerning their nature and action considerable knowledge has been acquired; but not a few morbid states are now known to arise from toxic conditions of autogenetic origin, and regarding these we are still in much ignorance. Dr. Allan MacFadyen, in a recent number of the *Polyclinic*, presents a convenient grouping of the more definitely recognised forms of auto intoxication:—(1) Those resulting from a failure of function in certain organs, with, as a consequence, non-neutralisation of poisonous metabolic products: myxœdema, pancreatic diabetes, acute yellow atrophy of the liver, and Addison's diseases are examples. (2) Those due to metabolic anomalies whereby intermediate metabolic products enter the circulation, as occurs in gout and diabetes. (3) Those resulting from the retention of metabolic products in various organs, as seen in the symptoms following severe burns, carbonic acid poisoning, and uræmia. (4) Those dependent on the over-production of physiological and pathological products, instances being acetonuria, cystinuria, and diabetic coma. Indeed, it would almost seem that every class of cell may be capable, should it depart from its normal function, of acting as a centre for the formation of products which may exert a deleterious influence on the organism as a whole. We have much still to learn regarding auto-intoxication, but doubtless a deeper study of pathology will show it to be an important factor in the production of many phases of disease the causation of which at present remains obscure.

